# Prenatal diagnosis of paternal uniparental disomy for chromosome 2 in two fetuses with intrauterine growth restriction

**DOI:** 10.1186/s13039-023-00647-z

**Published:** 2023-08-23

**Authors:** Xuemei Tan, Bailing Liu, Tizhen Yan, Xiaobao Wei, Yanfeng Qin, Dingyuan Zeng, Dejian Yuan

**Affiliations:** 1 Department of Medical Genetics, Liuzhou Municipal Maternity and Child Healthcare Hospital, Liuzhou, China; 2 Liuzhou Key Laboratory of Birth Defects Prevention and Control, Liuzhou Municipal Maternity and Child Healthcare Hospital, Liuzhou, China; 3 Liuzhou Key Laboratory of Thalassemia Prevention and Control, Liuzhou Municipal Maternity and Child Healthcare Hospital, Liuzhou, China; 4 Department of Medical Genetics, Liuzhou Hospital of Guangzhou Women and Children’s Medical Center, Liuzhou, China; 5 Department of Perinatal Health, Liuzhou Municipal Maternity and Child Healthcare Hospital, Liuzhou, China; 6 Department of Prenatal Diagnosis Center, Dongguan Maternal and Child Health Hospital, Dongguan, China; 7 Department of Gynecology, Liuzhou Municipal Maternity and Child Healthcare Hospital, Liuzhou, China

**Keywords:** Prenatal diagnosis, Paternal uniparental disomy, Whole-exome sequencing, Genomic imprinting, Intrauterine growth restriction

## Abstract

Uniparental disomy (UPD) is when all or part of the homologous chromosomes are inherited from only one of the two parents. Currently, UPD has been reported to occur for almost all chromosomes. In this study, we report two cases of UPD for chromosome 2 (UPD2) encountered during prenatal diagnosis. The ultrasound findings of the fetuses from two unrelated families showed intrauterine growth restriction. The karyotype analyses were normal. The two fetuses both had complete paternal chromosome 2 uniparental disomy detected by whole-exome sequencing, but their clinical outcomes were significantly different, with fetal arrest in case 1 and birth in case 2. In this report, we analyzed and discussed the phenotypes of the fetuses in these two cases and reviewed the literature on UPD2.

## Introduction

The concept of uniparental disomy (UPD) was first proposed by Engel [1] and refers to the phenomenon when all or part of an offspring’s homologous chromosome are derived from only the father or mother. UPD is a relatively rare chromosomal abnormality [2]. The pathogenic mechanism of UPD causing severe clinical phenotypes is mostly related to monogenic homozygous mutations or the disruption of imprinted gene expression [3, 4]. UPD on chromosomes 6, 7, 11, 14, 15, and 20 cause genomic imprinting disorders, resulting in disease [5]. For example, UPD15 leads to gene imprinting abnormalities in the 15q11q13 region, resulting in Prader-Willi/Angelman syndrome. Prader-Willi syndrome is a rare genetic disorder, characterized by clinical features including hypotonia and weakness, severe obesity, hypogonadism, and intellectual disability [6]. Patients with Angelman syndrome have clinical manifestations such as intellectual defects, microcephaly, ataxia, and seizures [7]. UPD of the X chromosome may lead to X-linked recessive disorders [8]. In UPD cases, the possibility of homozygous mutations in autosomal recessive genes is greatly increased. Diseases caused by this mechanism have been reported, such as LPS-responsive beige-like anchor deficiency, congenital ichthyosis, and Fanconi anemia [9–11]. The clinical phenotype of the fetus in the two cases reported here is closely associated with this mechanism.

With the development of genetic diagnostic techniques, more chromosomal abnormalities or genetic abnormalities have been detected during prenatal diagnosis, and most prenatal diagnostic techniques such as karyotyping, quantitative fluorescence PCR, copy number variant sequencing (CNV-Seq), and array comparative genomic hybridization cannot be used to screen for UPD [12, 13]. Using traditional genetic diagnosis methods, some cases of UPD may be overlooked [2]. Current genetic methods that can detect UPD with high accuracy include SNP arrays, short tandem repeat sequencing, and whole-exome sequencing (WES) [12, 13].

Here, we describe two pregnant women who had abnormal fetal development detected through ultrasound examination. Subsequent genetic diagnosis with WES found the presence of paternal uniparental disomy for chromosome 2. We analyze and discuss the inconsistent phenotypes of the fetuses in these two cases and review previous literature on UPD for chromosome 2 (UPD2).

## Case presentation

### Case 1

A 26-year-old pregnant woman presented to our hospital for prenatal diagnosis because the ultrasound results showed that the fetal limbs were abnormally short. She had given birth to a healthy baby boy two years before. She and her husband had no family history of congenital malformations, intellectual defects, or other genetic diseases. At 29 + 6 weeks gestation, the fetal ultrasound findings were as follows: biparietal diameter = 70 mm, − 2.55 SD; head circumference = 261 mm, − 1.99 SD; abdominal circumference = 247 mm, − 1.27 SD; femoral length = 46 mm, − 4.52 SD. Unfortunately, the fetal heartbeat stopped at 35 weeks + 1 day of gestation.

### Case 2

A 33-year-old pregnant woman came to our hospital for pregnancy examination late in her pregnancy, which was her first. The prenatal ultrasound results done at other hospitals showed that the fetal growth was low, but the fetal cardiac ultrasound was not abnormal. Subsequently, the prenatal diagnosis results suggested that the fetal chromosome karyotype was normal, and the SNP array results revealed chromosome 2 UPD with unknown parentage. The pregnant woman came to our department to clarify the genetic abnormality of fetus. At 34 weeks of gestation, the fetal ultrasound results showed the following: biparietal diameter = 80 mm, − 2.27 SD; head circumference = 288 mm, − 2.15 SD; abdominal circumference = 282 mm, − 1.49 SD; femoral length = 56 mm, − 3.56 SD. At 36 weeks + 6 days of pregnancy, the fetal membrane ruptured. Considering that the fetus had intrauterine growth restriction (IUGR) and might have difficulty in normal delivery, the female baby was delivered by cesarean section.

## Materials and methods

### Karyotype analysis

After informed consent was obtained, fetal cord blood was collected for chromosome karyotype analysis. First, conventional cell culture was performed according to the standard procedure. Then, the cells were treated with colchicine to obtain mitotic metaphase cells. Metaphase chromosome specimens were prepared after hypotonic and fixation and were stained with Giemsa for banding display. Thirty metaphase cells with a resolution of 500 bands were screened for karyotyping using the Zeiss automated chromosome analysis system.

### Genomic analysis

Parental peripheral blood samples and fetal amniotic fluid were collected after informed consent was obtained. Genomic DNA was extracted and purified using the QIAamp DNA Midi Kit (QIAGEN, Crawley, UK) according to the manufacturer’s instructions. Library preparation was performed, followed by whole-exome sequencing on the Illumina HiSeq 2500 platform (Illumina, CA, USA). After sequencing data was obtained, the sequence alignment was performed with reference to the human genome build GRCh37. GATK (v.3.8) software was used for single nucleotide variant (SNV) and insertion deletion (indel) detection. ANNOVAR software was used to annotate the variants. The annotated data from WES were screened: first, it was compared with the 1000 Genomes Project and Genome Aggregation Database to eliminate relatively common genetic variation (minor allele frequency-MAF > 1%) in the population. Then, variants predicted by Variant Effect Predictor that have only mild, moderate, and modifying effects on the phenotype and are located at non-coding regions were removed. Multiple algorithms were then used to make predictions about the deleteriousness of the remaining variants. These included SIFT, PolyPhen, ClinPred, and Mutation Taster. The mutations predicted as “harmful” and “possibly harmful” were retrieved from HGMD and Clinvar to enrich for and classify rare and potentially pathogenic mutations.

CNV-seq mainly consists of three steps: (1) Genomic DNA extraction: Genomic DNA (15 ng) was extracted from amniotic fluid cells and fragmented. (2) Library construction, purification and quality control: the DNA ends of small fragments were complemented by adding adenine deoxyribonucleotides and then ligated with the universal primer by TA ligation for sequencing to form a DNA library. The libraries were then quality controlled using quantitative fluorescent PCR. The library needed a concentration greater than 10 pmol/L, a melting temperature of 78–81 °C, and normal melting peaks without dimerization or broad peaks to pass QC. (3) Sequencing: Sequencing was performed on the Nextseq CN500 (Illumina). Approximately 5 million single-end reads of 45-bp in length were obtained. Approximately 2.8–3.2 million accurately located 36-bp reads were aligned to the human reference genome for each sample using the Burrows–Wheeler alignment algorithm and were then allocated into 20-kb bins on each of the 24 chromosomes. Then, the log2 value of the average CNV along the length of each sequencing bin on each chromosome was calculated. A log2 value of 0 represents two copies (normal), A log2 value of 1.5 represents three copies (duplication), and a log2 value of 0.5 represents one copy (deletion). The average chromosomal copy number of triploids can range from 2.05 (5%) to 2.95 (95%), while the average chromosomal copy number of monoploids can range from 1.05 (5%) to 1.95 (95%). Finally, the pathogenicity of candidate variants was evaluated according to the guidelines of the American College of Medical Genetics and Genomics (ACMG).

## Results

**Fig. 1:**
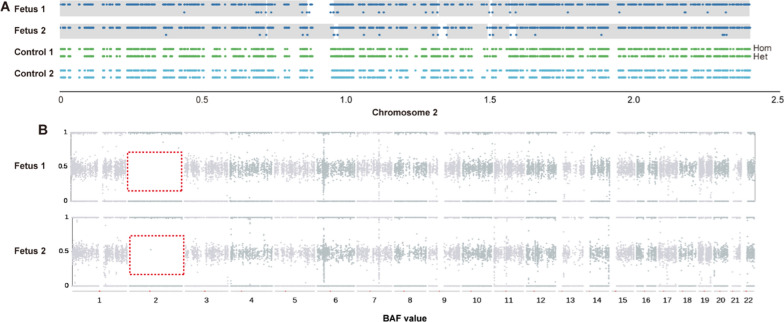
**A** There were no heterozygous SNPs on chromosome 2 in the genotypes of fetus 1 and fetus 2. The x-axis shows the number of bases of chromosome 2 (in units of 1e8 bp). “Hom” represents homozygote and “Het” represents heterozygote. **B** The B-allele frequency within the red dashed box should have a distribution with a value of 0.5. However, there is an obvious absence of the 0.5 value, with only two values (0/1) present, indicating that there has been a run of homozygosity

**Fig. 2:**
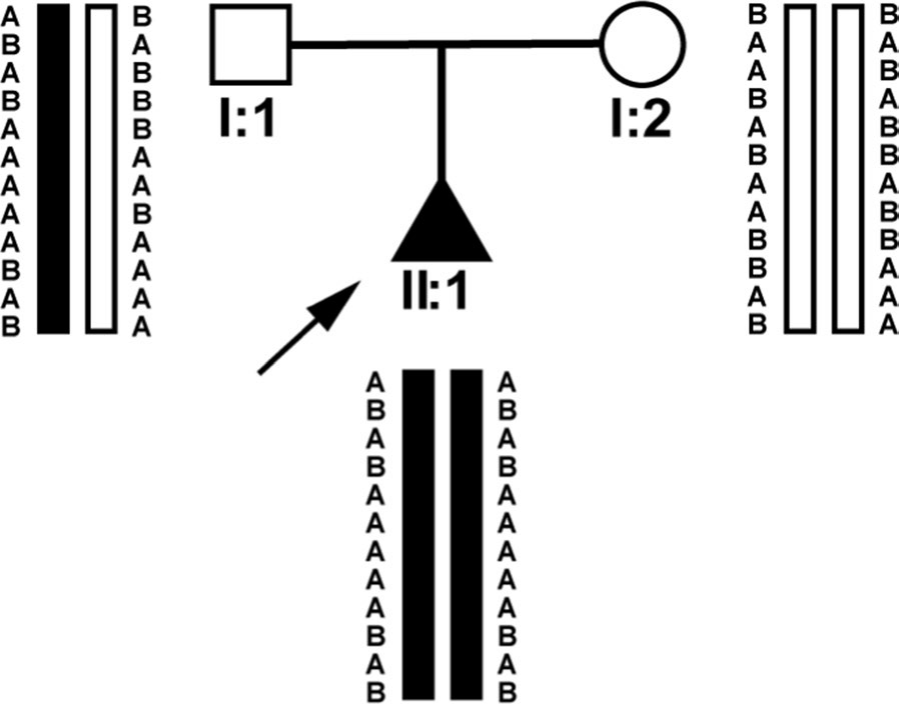
Here, a schematic diagram was used to show that by using WES, we found that the homozygous loci of chromosome 2 in fetus 1 and fetus 2 were always consistent with the father, indicating that their UPD2 was of paternal origin

The ultrasound findings in the case 1 and 2 fetuses suggested significantly less fetal development than normal fetuses at the same age, with normal fetal karyotypes (46, XN) and no chromosomal number or structural abnormalities. CNV-seq did not show any copy number variants. The SNP genotyping results showed that there were runs of homozygosity region on chromosome 2 with a normal copy number of two (Fig. 1). Meanwhile, WES sequencing data analysis revealed that the fetal genotypes remained consistent with the father at the homozygous locus of chromosome 2 (Fig. 2). This suggested paternal UPD throughout chromosome 2. We analyzed and filtered the variants detected by WES on chromosome 2 with the following selection criteria: (a) novel or rare (MAF < 1%) variants, including missense, loss-of-function (such as frameshift, nonsense) and splice site variants with potential deleterious effects; (b) variants are heterozygous in the father and homozygous in the fetus; (c) variants follow an autosomal recessive inheritance pattern; (d) and are described in the OMIM database. In case 1, two rare missense variants were found on chromosome 2 in the fetus. Table 1 summarizes the homozygous loci on chromosome 2 that may be associated with the clinical phenotype of the case 1 fetus. Four rare homozygous nonsynonymous variants were identified on chromosome 2 in case 2 fetus. Table 2 summarizes the homozygous loci on chromosome 2 of the case 2 fetus. The harmfulness prediction results of these variants are also displayed.

**Table 1:** Details of the homozygous loci on chromosome 2 of fetus 1

Gene	Location	Variant	Zygosity			Pathogenicity prediction				GnomAD (MAF %)
			Fetus	Father	Mother	SIFT	Poly-Phen	Clin-Pred	Mutation Taster	
*TTN*	chr2:179,577,553	c.27199G > A(p.Val9067Met)	Hom	Het	Wild	Tol	Beni	Tol	Poly	SA: 0.009808
										Lat: 0.002892
*IFT172*	chr2:27,683,894	c.2509G > A(p.Ala837Thr)	Hom	Het	Wild	Del	Prob dam	Del	Dis Caus	EA: 0.005437
										SA: 0.003266

*Hom* Homozygous, *Het* Heterozygous, *Wild*: Wild-type, *Del* deleterious, *Tol* tolerated, *Prob dam* probably damaging, *Poss dam* possibly damaging, *beni* benign, *Dis Caus* disease causing, *Poly* polymorphism, *Neu* neutral

Higher GERP scores are more deleterious

The gnomAD MAF: *NFE* non-Finnish European, *Lat* Latin, *Afr* African, *EA* East Asian; *Fin* Finish, *SA* South Asian

**Table 2:** Summary of the homozygous loci found on chromosome 2 of fetus 2

Gene	Location	Variant	Zygote			Pathogenicity prediction				GnomAD (MAF %)
			Fetus	Father	Mother	SIFT	Poly-Phen	Clin-Pred	Mutation Taster	
*TTN*	chr2:179,392,289	c.107564G > A(p.Ser35855Asn)	Hom	Het	Wild	Tol	Beni	Del	Poly	–
*MMADHC*	chr2:150,432,353	c.481 T > A(p.Phe161Ile)	Hom	Het	Wild	Del	Poss dam	Tol	Dis Caus	EA: 0.1220
*ATP6V1B1*	chr2:71,190,308	c.926A > G(p.Glu309Gly)	Hom	Het	Wild	Del	Beni	–	Dis Caus	EA: 0.4261 Fin: 0.003981
										SA: 0.003266
*VWA3B*	chr2:98,709,718	c.163A > T(p.Ile55Phe)	Hom	Het	Wild	Del	Prob Dam	Del	Poly	NFE: 0.001554
										EA: 0.005119

*Hom* Homozygous, *Het* Heterozygous, *Wild* Wild-type, *Del* deleterious, *Tol* tolerated, *Prob dam* probably damaging, *Poss dam* possibly damaging, *beni* benign, *Dis Caus* disease causing, *Poly* polymorphism, *Neu* neutral

Higher GERP scores are more deleterious

The gnomAD MAF: *NFE* non-Finnish European, *Lat* Latin, *Afr* African, *EA* East Asian, *Fin* Finish, *SA* South Asian

## Discussion

UPD is caused by chromosome segregation errors during meiosis and/or mitosis, which can occur independently or jointly [14, 15]. The four main formation mechanisms of UPD are often described as monosomic rescue, trisomic rescue, postfertilization division errors, and gamete complementation [5, 14]. UPD can sometimes cause homozygous mutations in recessive genes or imprinted gene expression disorders, leading to serious clinical consequences such as rib dysmorphism, mental retardation, or short stature [16–18]. So far, UPD has been reported on almost all chromosomes. Cases of UPD2 are less frequently reported [19]. Some of the UPD2 cases reported to date were found in children with developmental defects or mental abnormalities. Pathogenic mutations located on chromosome 2 were detected. UPD2 resulting in homozygosity of pathogenic mutations leads to certain diseases such as Catel-Manzke syndrome/VCRL syndrome, infantile hypotonia with psychomotor retardation and characteristic facies 2, and hepatocerebral mitochondrial DNA depletion syndrome [3, 20, 21]. Some UPD2 cases were discovered in normal individuals during paternity testing [2, 22, 23].

In this report, we conducted a study on two fetuses with abnormal intrauterine development. The limbs of fetus 1 and fetus 2 were significantly shorter than those of normal fetuses of the same age. Unfortunately, fetus 1 was stillborn, and fetus 2 was born prematurely. The results of karyotyping and CNV-seq showed no numerical or structural abnormalities of the chromosomes. The presence of chromosomal chimerism was also excluded. Paternal UPD2 in the fetuses by WES. We performed WES to look for potentially pathogenic mutations. Some variants that may be associated with IUGR were screened by WES, such as the *TTN* variants. *TTN* variants have been reported to be linked to multiple skeletal and cardiac myopathies [24]. Genetic analysis results revealed that the *TTN* missense variants in case 1 and case 2 were inherited from the father and were homozygous due to UPD of chromosome 2. However, *TTN* truncating variants are a common cause of dilated cardiomyopathy, however their penetrance for dilated cardiomyopathy in general populations is low [25, 26]. The *TTN* variants and other variants detected by WES were considered as variants of uncertain significance according to the guidelines of the ACMG. Considering that the fetuses’ parents were both healthy individuals and combined with the ACMG score, no potential pathogenic mutations were found. We consider that the variants detected by WES are unlikely to be involved in the intrauterine abnormal development phenotypes of the fetuses in both cases. Additionally, we speculate that there may be an imprinting region on chromosome 2 that may lead to developmental disorders or intellectual problems. UPD2 would cause this imprinting region to not be expressed, thus leading to abnormal embryonic development. The effects caused by this genetic imprinting region may potentially vary in penetrance across different populations.

This is the first report of UPD2 found in fetuses with IUGR that were eventually stillborn. After excluding genomic pathogenic mutations, we speculated whether there might be potential maternal imprinting regions on chromosome 2. Paternal UPD2 prevents the expression of the imprinting region and thus affects normal development of the embryo. There are currently no reports of genetic imprinting regions on chromosome 2 [2, 27]. It is possible that an imprinting region on chromosome 2 has not been discovered yet or has been overlooked due to the mild phenotype of the patients. We summarized the homozygous variants on chromosome 2 in fetuses 1 (Table 1) and fetus 2 (Table 2). Since we filtered out genes not included in OMIM, further studies may be conducted in the future to explore the functions of the filtered genes and determine whether they are related to the phenotypes observed in these two cases. Further validation experiments are needed to confirm whether there is an imprinting region on chromosome 2. Furthermore, our study shows that UPD might not be identified through traditional prenatal diagnosis. With the advantages of high resolution and throughput, WES technology can be used to monitor the presence of UPD in the case of microduplications, microdeletions, or single gene point mutations. The combination of WES with cytogenetics and CNV-seq has greatly improved prenatal diagnostics.

## Data Availability

The datasets for this article are not publicly available due to concerns regarding participant/patient anonymity. Data for this study can be requested from the corresponding author.
